# Postpartum Spinal Epidural Lipomatosis With Epidural Venous Engorgement

**DOI:** 10.7759/cureus.45184

**Published:** 2023-09-13

**Authors:** Abdalhai Alshoubi

**Affiliations:** 1 Anesthesiology and Critical Care, University of Illinois College of Medicine Peoria, Peoria, USA

**Keywords:** decompressive laminectomy, mri images, leg weakness, epidural venous engorgement, spinal epidural lipomatosis

## Abstract

Spinal epidural lipomatosis (SEL) refers to a condition characterized by the abnormal growth of fatty tissue within the vertebral canal, situated outside the spinal canal itself. This expansion of fat can result in symptoms such as back pain and radiculopathy. The majority of cases remain without noticeable symptoms. However, when SEL does cause symptoms, it is frequently linked to using external steroids. The contributing factors to SEL include obesity and Cushing's syndrome. The presentation of SEL can mimic other spinal disorders such as epidural hematoma, spinal stenosis, and degenerative joint disease. Patients might present with gradually progressing and long-standing complaints of back pain, muscle weakness, numbness, loss of bladder or bowel control, lack of coordination, abnormal reflexes, and, in rare instances, paralysis.

We are reporting a case involving a 34-year-old female with obesity, who experienced sudden weakness in her left lower extremity shortly after a recent uncomplicated vaginal delivery using epidural analgesia. A magnetic resonance imaging (MRI) of her thoracic (T) and lumbar spine revealed spinal cord compression secondary to extensive posterior epidural lipomatosis associated with epidural venous engorgement. The patient underwent an immediate laminectomy procedure at the T3, T5, T7, and T9 levels to alleviate the compression. Postoperatively, she underwent a course of physiotherapy and gradually regained her normal muscle strength. She was discharged in stable condition on the sixth day after the surgery.

## Introduction

Spinal epidural lipomatosis (SEL) is an infrequent medical condition characterized by the abnormal growth of unencapsulated fatty tissue in the extradural space [1]. This pathological progression leads to the constriction of the spinal canal and the subsequent compression of nearby neural structures. The symptoms emerge as a result of the compression of nerve tissue and encompass myelopathy, radiculopathy, neurogenic claudication, loss of sensory perception, difficulties with urination, weakness in the lower extremities, and, in rare instances, cauda equina syndrome [2].

The precise underlying mechanisms of SEL remain elusive; however, it appears to be associated with prolonged steroid usage, obesity, excessive production of endogenous steroids, or surgical interventions. Treatment strategies typically lean toward conservative measures and focus on addressing the root cause. Weight loss is consistently beneficial. For individuals experiencing severe symptoms, surgical intervention might be necessary [3].

## Case presentation

A 34-year-old female, gravida 8, para 2 with obesity but no significant prior medical issues, presented to our hospital at 39 weeks and four days of gestation while in early labor. Her height is 154.9 cm, and her weight is 108.9 kg, resulting in a body mass index of 46 kg/m².

The patient requested an epidural labor analgesia. Using the loss of resistance to saline technique, an 18-gauge Tuohy needle was used to locate the epidural space at the lumbar 4-5 interspace, at a depth of 8 cm. The epidural catheter was then inserted with ease while the patient was seated, threaded to the 15 cm mark, and secured with the catheter end positioned at the skin's 13 cm mark after removing the needle. This left 5 cm of the catheter within the epidural space. Following a test dose of 3 mL of 2% lidocaine with 1:200000 epinephrine, ensuring that there were no signs of intravascular or subarachnoid cannulation, the catheter was fixed in place. The catheter was then connected to an infusion of bupivacaine 0.125% and fentanyl 2 μg/mL at a rate of 7 mL/hour. Throughout her labor, which lasted approximately four hours, the patient remained comfortable and stable.

Following a vaginal delivery, the catheter was successfully removed without any complications. The patient's postnatal recovery was uneventful until two days after the delivery when she reported severe back pain in the thoracic (T) and lumbar regions along with left leg weakness. She denied any numbness or weakness in her upper extremities or right lower extremity, as well as any urinary retention or incontinence.

Her vital signs were within normal limits, and physical examinations of her heart, lungs, and abdomen revealed no abnormalities. No rashes, skin lesions, or hematomas were observed on her back. Neurologically, her mental status, speech, comprehension, and cognitive function were all intact without any signs of neglect or visual field impairment. Cranial nerve testing yielded normal results, and no signs of papilledema or optic atrophy were noted. Motor examination revealed grade 4/5 strength in her left lower extremity, which includes knee extension and knee flexion, and grade 3/5 strength in ankle dorsiflexion and ankle plantar flexion. Left hip flexion and extension strength was 5/5. The motor examination of the right lower extremity was unremarkable.

No notable differences in muscle tone were observed between sides, and coordination tests, such as the heel-shin slide, showed normal results. Reflexes were brisk but not excessively hyperactive, remaining consistent with those on the unaffected side. Both clonus and the Babinski reflex were negative bilaterally. Sensory examination revealed no loss of light touch in dermatomal or non-dermatomal areas when tested with a cotton wool bud.

**Figure 1:**
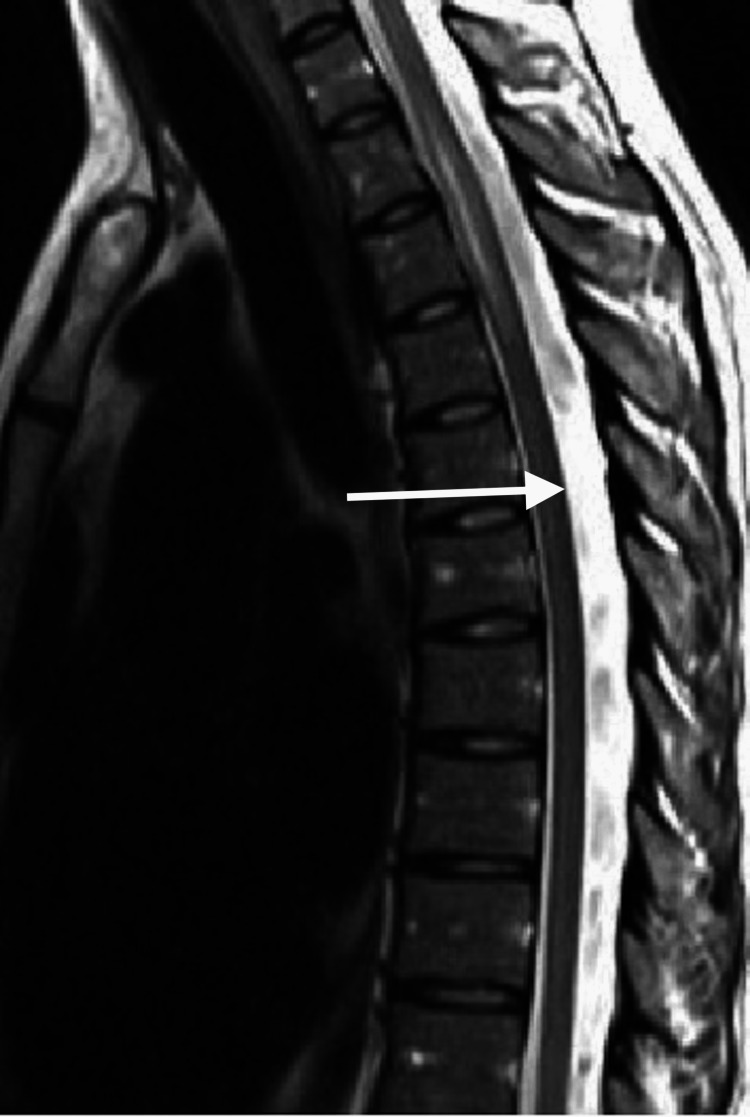
Normal sagittal T1-weighted MRI of the thoracic spine shows epidural fat (white arrow), and it leads to neither any compression nor the displacement of the dural sac MRI: magnetic resonance imaging

**Figure 2:**
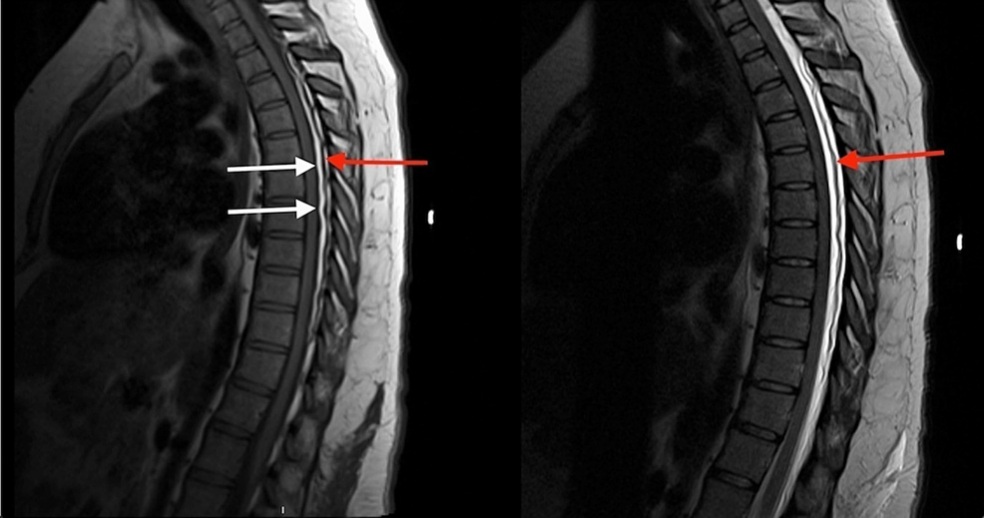
Sagittal T1-weighted MRI (left) and T2-weighted MRI (right) show a thick proliferation of epidural fat (white arrows) compressing and displacing the dural sac toward the anterior and epidural vein engorgement (red arrows) MRI: magnetic resonance imaging

MRI scans with T1-weighted and T2-weighted images disclosed hyperintense collections within the posterior subdural and epidural spaces spanning from thoracic (T) 2 to thoracic 11 levels, as well as epidural venous engorgement (Figures 1, 2).

The patient was taken to the operating room under emergency. A midline skin incision was made extending from T2 to T10 followed by the dissection of paraspinal muscles; skipped laminectomy was performed at T3, T5, T7, and T9. Wide neural decompression was performed. Severe lipomatosis and mass effect in addition to a large ingurgitated epidural vein were identified. No significant hematoma was found. The patient tolerated the surgery without any complications. Postoperatively, the patient underwent physiotherapy, and eventually, she regained her normal strength and was discharged home in stable condition on postoperative day 6.

## Discussion

Spinal epidural lipomatosis (SEL) arises from the abnormal accumulation of adipose tissue outside the spinal canal, potentially resulting in mass effect or venous engorgement, which can compress the spinal cord or the nerve roots [1].

While SEL is typically localized posterior to the spinal cord in the thoracic (T4-T8) or lumbar (L4-L5) vertebral region, the distribution of adipose tissue deposition varies based on the underlying cause of the condition. A study by Fogel et al. revealed that the majority of SEL cases caused by exogenous steroids primarily affect the thoracic spine. Conversely, endogenous steroid-related SEL tends to involve both thoracic and lumbosacral areas to a similar extent. Obesity-related SEL and idiopathic cases predominantly lead to lumbosacral involvement [2].

Multiple risk factors contribute to SEL, with steroid medications being the primary cause [2]. Chronic steroid use triggers the stimulation of glucocorticoid receptors in adipose tissue, leading to hypertrophy. Clinical conditions such as Cushing's syndrome, carcinoid tumors, and hypothyroidism, associated with the overproduction of endogenous steroids, have also been linked to SEL [3]. Obesity is another risk factor due to the chronic inflammation and hypertrophy of adipose tissue within the spinal canal. SEL has even been reported in individuals with a history of spine surgery, while in some cases, no clear cause can be identified [4-6]. In our patient, obesity in addition to postpartum epidural venous engorgement was thought to be the driving factor.

Clinical presentation often parallels that of other spinal cord compression disorders. SEL symptoms are nonspecific and can manifest as myelopathy, radiculopathy, sensory disturbances, or claudication. While some experience back pain, weakness, paresthesias, or ataxia early on, others remain asymptomatic until the disease reaches advanced stages [5]. Rarely, cauda equina syndrome and acute paraplegia may be initial signs [6]. The diverse symptom range is partly due to variable adipose accumulation across different spinal canal regions. Symptoms may develop rapidly, but in most instances, they evolve over months to years [7]. SEL typically shows a higher incidence in males than females. Although uncommon, SEL has been documented in children [8].

Treatment mainly is conservative, including weight loss and addressing the underlying cause [7]. Surgical decompression is indicated for patients with severe symptoms or those who do not respond to conservative management [9].

## Conclusions

The clinical manifestation of SEL frequently resembles that of other spinal cord compression disorders, including conditions such as epidural hematoma. MRI serves as the definitive diagnostic tool. Initially, a conservative approach is favored for management, while surgical decompression becomes necessary for severe neurological deficits. In our specific case, the clinical presentation of SEL was characterized by the engorgement of the epidural vein.
